# Systematic Analysis of the Lysine Crotonylome and Multiple Posttranslational Modification Analysis (Acetylation, Succinylation, and Crotonylation) in Candida albicans

**DOI:** 10.1128/mSystems.01316-20

**Published:** 2021-01-26

**Authors:** Xiaowei Zhou, Nana Song, Dongmei Li, Xiaofang Li, Weida Liu

**Affiliations:** a Department of Medical Mycology, Institute of Dermatology, Chinese Academy of Medical Sciences & Peking Union Medical College, Nanjing, China; b Jiangsu Key Laboratory of Molecular Biology for Skin Diseases and STIs, Nanjing, China; c Department of Microbiology and Immunology, Georgetown University Medical Center, Washington, DC, USA; d Center for Global Health, School of Public Health, Nanjing Medical University, Nanjing, China; NYU School of Medicine

**Keywords:** *Candida albicans*, crotonylome, histone, lysine crotonylation motif, PPI analysis

## Abstract

Candida albicans is an opportunistic pathogen that causes lethal fungal infections in immunocompromised patients. Lysine crotonylation is a newly discovered PTM (posttranslational modification) epigenetic type that may play a critical role in regulating gene expression. In this study, we used an antibody-enrichment approach along with LC-MS/MS to carry out a quantitative crotonylome analysis in C. albicans. We found a total of 5,242 crotonylation sites and 1,584 crotonylated proteins among 9,038 proteins in this organism. Of these crotonylated proteins, a few unique crotonylated motifs are noted such as D and E in positions +1, +2, or +3 or K and R in positions +5 or +6, while A, E, F, G, P, W, and Y are in the −1 position or A, K, and R are found in positions −5, −6, −7, or −8. Functional analysis has shown that a majority of the crotonylated proteins are related to biosynthetic events and carbon metabolism. When combined with previously collected data on acetylation and succinylation, PPI (protein-protein interaction network) analysis reveals that proteins with functions in ribosomal biogenesis, oxidative phosphorylation, nucleus activity, and proteasome formation are heavily modified by these three PTM types. To the best of our knowledge, this is the first crotonylome study carried out in C. albicans and is an important step to a better understanding of the biological and pathogenic impact of PTM in C. albicans.

**IMPORTANCE** C. albicans is a kind of pathogen of fungal infections that is found worldwide. Lysine crotonylation of proteins as a recently discovered PTM (posttranslational modification) may have a critical role in regulating cells. We first carried out large-scale analysis of crotonylated proteome and multiple PTM analysis (acetylation, succinylation, and crotonylation), then drew a diagram to show multiple PTM sites on histones in C. albicans of our study. This study about crotonylome in human pathogenic fungi is a milestone that first and deeply investigates the functional analysis of crotonylated proteins in C. albicans, which marks an important start for further research.

## Abstract (precis)

C. albicans is a kind of pathogen of fungal infections that is found worldwide. Lysine crotonylation of proteins as a recently discovered PTM (posttranslational modification) may have a critical role in regulating cells.

## INTRODUCTION

Candida albicans is a commensal organism in the healthy human gut, but it can cause systemic fungal infections in certain patients (1). Mucosal sites such as the mouth, gut, vaginal membrane, and the skin are the most commonly affected sites of explosive growth, and severe invasive infection with high fatality is found in immunocompromised patients (2). In the survey “The Extended Prevalence of Infection in Intensive Care” (EPIC II), fungal organisms account for 19% of the positive isolates. Of these, the most common fungal organisms are *Candida* spp. (17%) (3), and C. albicans accounts for 70% of those *Candida* spp. The data of EPIC II also reveal that patients with *Candida* bloodstream infections (BSIs) place a particularly heavy burden on health care resources (4). In a similar study in a NICU (neonatal intensive care unit) located in western China, the overall incidence of candidemia was 1.4% (69 over 5,075 neonates), and the proportion of C. albicans infections among those neonates with candidemia was 43.5% (5). Moreover, the resistant fungal isolates commonly found in clinical settings and the overall increase in the immunocompromised population (given the increased prevalence of advanced anticancer chemotherapies) also present greater challenges for control of systemic infections. The conversion of C. albicans between its commensal and pathogenetic phases is still not well understood.

The word “epigenetics” (which includes protein posttranslational modifications [PTMs]) means external modifications to DNA that affect gene expression without altering the DNA sequence. Compared with “classic” DNA epigenetics, PTM affects gene expression through amino acid residues such as lysine in histone. Recently, novel therapies based on targeting various epigenetic mechanisms have been applied under a variety of disease and immunological conditions such as autoimmunity (6), cancer (7, 8), neurodegenerative disease (9), and immunological disorders (10). With specific regard to PTM, the protein can be modified by acetylation, phosphorylation, succinylation, crotonylation, methylation, butyrylation, propionylation, glutarylation, etc. Presumably, all these PTMs could be found to play some role in the regulation of gene expression in microorganisms (11, 12). In our previous studies, we have identified acetylation (Kac) (13) and succinylation (Ksuc) (14) modifications in C. albicans.

Lysine crotonylation (Kcr) is an acyl modification that may prove to be distinct in its functionality from Kac and Ksuc. Kcr often marks either active promoters or enhancers of genes in both human somatic and mouse male germinal cells (15). The YEATS domain of histone crotonylation at H3K9cr has been related to virulence-related biology of C. albicans (16). In male germinal cells, Kcr is enriched on sex chromosomes for testis-specific genes and indicates its significant role driving male haploid cell gene expression (17). Histone crotonylation also abounds in the intestinal epithelium, especially the crypt fraction of the small intestine and the colon, and may be promoted by the short-chain fatty acids (SCFAs) that are generated by intestinal microbiota (18). Protein function is deeply affected by PTMs such as histone lysine crotonylation (Kcr). With discoveries of novel types of PTM, the biological significance of PTM becomes increasingly believable; however, the functional specificity of each PTM type and the substrate proteins under each PTM regulation are not well understood, although different forms of PTM may be catalyzed by the same enzyme. For example, two homologous lysine acetyltransferases (KATs), p300 and CBP, have been noted for their action on acetylation, propionylation, butyrylation, succinylation, glutarylation, and crotonylation (19–23). Lysine deacetylases (KDACs) such as sirtuins (SIRT1 to 3) may participate in or even catalyze crotonylation (24). The histone deacetylase (HDAC; classes I, IIa, and IIb) inhibitor SAHA (suberoylanilide hydroxamic acid) also inhibits nonhistone and histone protein decrotonylation in A549 (25).

In this study, we use anticrotonylation antibody-enrichment technology and high-resolution liquid chromatography-mass spectrometry (LC-MS/MS) to analyze crotonylation in C. albicans. With this approach, we have demonstrated that lysine crotonylation is quite prevalent in C. albicans and is marked by a total of 5,242 crotonylation sites within 1,584 proteins during the exponential growth phase of the SC5314 strain. The functions of substrate proteins under crotonylation were classified, and local amino acids sequences around the crotonylated lysine sites were analyzed. Compared with previously published (13) and succinylated proteins (Ksuc) (14) of profiles of the same strains, we perform a comprehensive analysis on lysine PTM pattern during the exponential growth phase of C. albicans using protein-protein interaction (PPI) network analysis.

## RESULTS

### 5,242 sites of 1,584 crotonylated proteins of C. albicans were detected.

**FIG 1:**
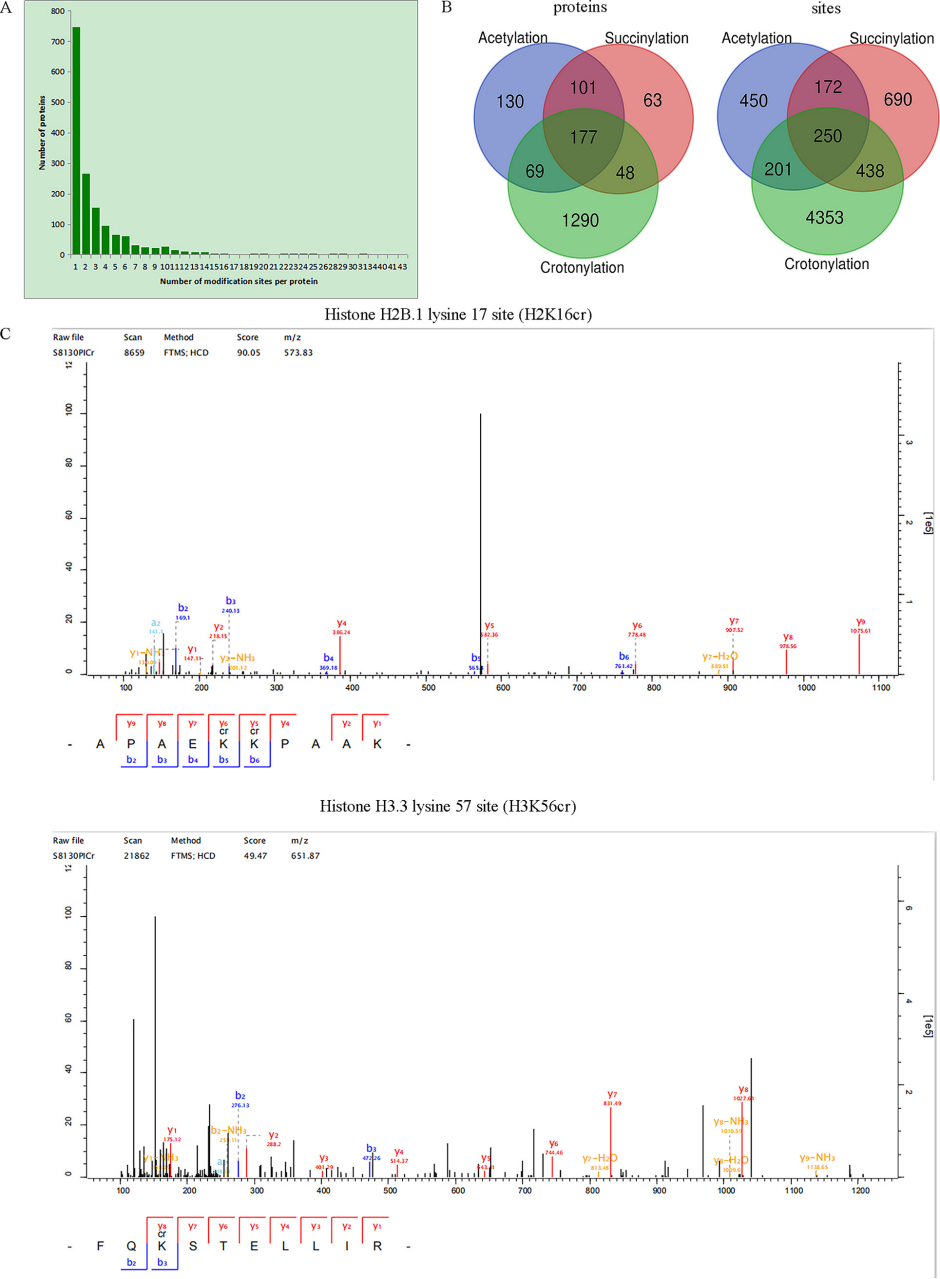
Quantitative crotonylation analyses of C. albicans. (A) The statistics of lysine crotonylation points identified in C. albicans proteins. (B) Venn diagrams show lysine crotonylation (Kcr), acetylation (Kac), and succinylation (Ksuc) analysis of C. albicans identified in this study and our previous study. (C) Identification and verification of lysine crotonylation.

To determine the level of crotonylation prevalence in C. albicans, we analyzed enriched crotonylated peptides of SC5314 using LC-MS/MS search tools for resulting MaxQuant data. We identified 5,242 crotonylation sites within 1,584 proteins, which account for 17.5% (1,584/9,038) of the entire C. albicans proteome. Of these 1,584 crotonylated proteins, 748 (47.2%) contain only a single crotonylated site (Fig. 1A), 267 (16.9%) contain two sites, and 154 (9.7%) have three crotonylation sites. Sixty-six proteins (4.2%) are found to have more than 10 crotonylation sites. For example, heat shock protein 90 (HSP90) bears 41 crotonylation sites (Table S1), and the homolog of HSP90 has been associated with heat stress adaptation and protein degradation.

10.1128/mSystems.01316-20.1 TABLE S1 Annotation_combine. Download Table S1, XLSX file, 1.9 MB. Copyright © 2021 Zhou et al. 2021 Zhou et al. This content is distributed under the terms of the Creative Commons Attribution 4.0 International license.

Compared with Kac and Ksuc prevalence in our earlier studies (13, 14), we found more crotonylation modification sites/proteins (3- to 5-fold higher) than in either Kac or Ksuc PTM form in C. albicans. The quantitative analysis of these three PTM forms also found that 250 crotonylation sites within 177 crotonylated proteins were also simultaneously modified by succinylation and acetylation (Fig. 1B).

### Functional analysis of lysine crotonylation in C. albicans.

The effects of crotonylated proteins on cellular processes at different subcellular locations were then further analyzed by Gene Ontology (GO) annotation and KOG (Clusters of orthologous groups for eukaryotic complete genomes) analysis.

GO annotation showed that the identified proteins were involved in various biological processes as shown in Table 1. The number of crotonylated proteins involved in the top three biological process groups were as follows: 792 proteins in the cellular process category (50%), 849 proteins in the metabolic process category (53.6%), and 529 proteins in the single-organism process category (33.4%). These crotonylated proteins were located, in descending order, in cell (718 proteins, 45.3%), in organelle (477 proteins, 30.1%), in macromolecular complex (314 proteins, 19.8%), and in membrane (300 proteins, 18.9%). The molecular functions associated with these crotonylated proteins break down as follows: 757 proteins are associated with binding (47.8%), 723 proteins with catalytic activity (45.6%), and 102 proteins with structural molecular activity (6.4%).

**TABLE 1:** Identified crotonylated protein distribution in GO

GO terms level 1	GO terms level 2	No. of proteins
Biological process	Metabolic process	849
	Cellular process	792
	Single-organism process	529
	Localization	186
	Biological regulation	161
	Cellular component organization or biogenesis	127
	Response to stimulus	119
	Multiorganism process	57
	Growth	51
	Other	42
Cellular component	Cell	718
	Organelle	477
	Macromolecular complex	314
	Membrane	300
	Membrane-enclosed lumen	66
	Extracellular region	25
	Other	5
Molecular function	Binding	757
	Catalytic activity	723
	Structural molecule activity	102
	Transporter activity	67
	Molecular function regulator	29
	Electron carrier activity	21
	Other	29

**FIG 2:**
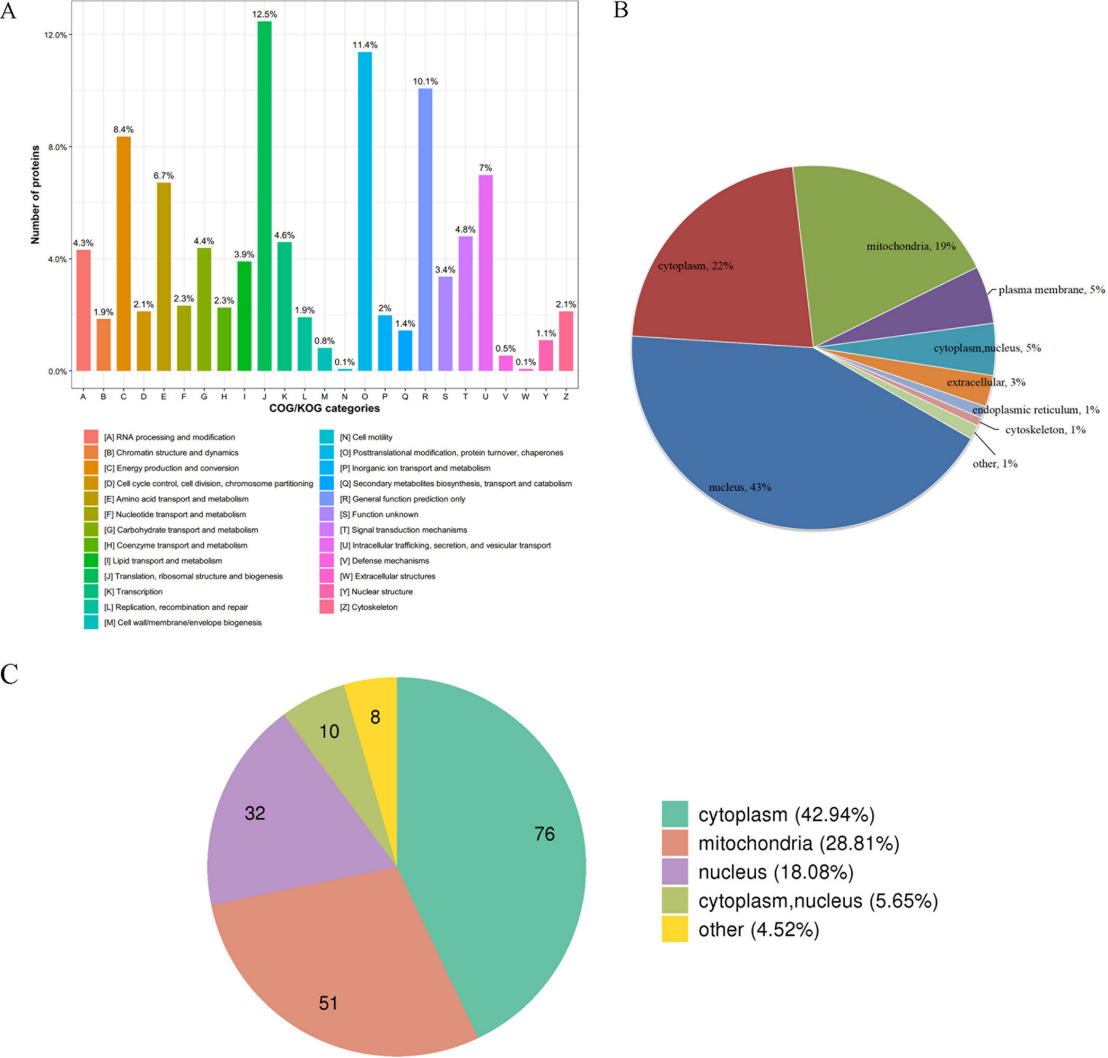
Classification of crotonylated proteins by KOG and subcellular location annotation information. (A) Identified crotonylated protein distribution in KOG category distribution. (B) Subcellular localization of identified crotonylated proteins. (C) Subcellular localization of identified crotonylated proteins bearing the three modifications.

The function classification of identified crotonylated proteins was then carried out with KOG analysis. We found that 182 proteins are associated with biogenesis, ribosomal structure, and translation (12.5%); 166 proteins with chaperones, posttranslational modification, and protein turnover (11.4%); and 147 proteins with general function prediction only (10.1%). The only other two categories with more than 100 identified proteins were as follows: 122 proteins (8.4%) in energy production and conversion and 102 proteins (7.0%) in secretion, intracellular trafficking, and vesicular transport. Consistent with the high level of proteins in energy production and conversion, we find that 98 proteins (6.7%) are related to amino acid transport and metabolism as shown in Fig. 2A and Table S2.

10.1128/mSystems.01316-20.2 TABLE S2 KOG_classify. Download Table S2, XLSX file, 0.4 MB. Copyright © 2021 Zhou et al. 2021 Zhou et al. This content is distributed under the terms of the Creative Commons Attribution 4.0 International license.

Inferred subcellular locations of identified crotonylated proteins are shown in Fig. 2B and Table S3 (identity classification). In the pie diagram of Fig. 2B, 676 crotonylated proteins are located in the nucleus (42.7%), 351 proteins are cytoplasmic proteins (22.2%), and 310 proteins are mitochondrial proteins (19.6%). The remaining proteins with portions of 5% or more include 81 proteins in plasma membrane (5.1%) and 73 proteins in both cytoplasm and nucleus (5%) locations. The top three subcellular locations of those crotonylated proteins described above agree with earlier results on the other two PTM forms (acetylation and succinylation). However, as shown in Fig. 2C and Table S3 (three modifications), the order is now different. Of a total of 177 proteins with three PTMs, 76 are located in the cytoplasm (42.94%), 51 in the mitochondria (28.81%), and 32 in the nucleus (18.08%). These results suggest that crotonylation may be more important for nuclear proteins than either acetylation or succinylation.

10.1128/mSystems.01316-20.3 TABLE S3 Subcellular_classify. Download Table S3, XLSX file, 0.02 MB. Copyright © 2021 Zhou et al. 2021 Zhou et al. This content is distributed under the terms of the Creative Commons Attribution 4.0 International license.

### Functional enrichment analysis of lysine crotonylation in C. albicans.

**FIG 3:**
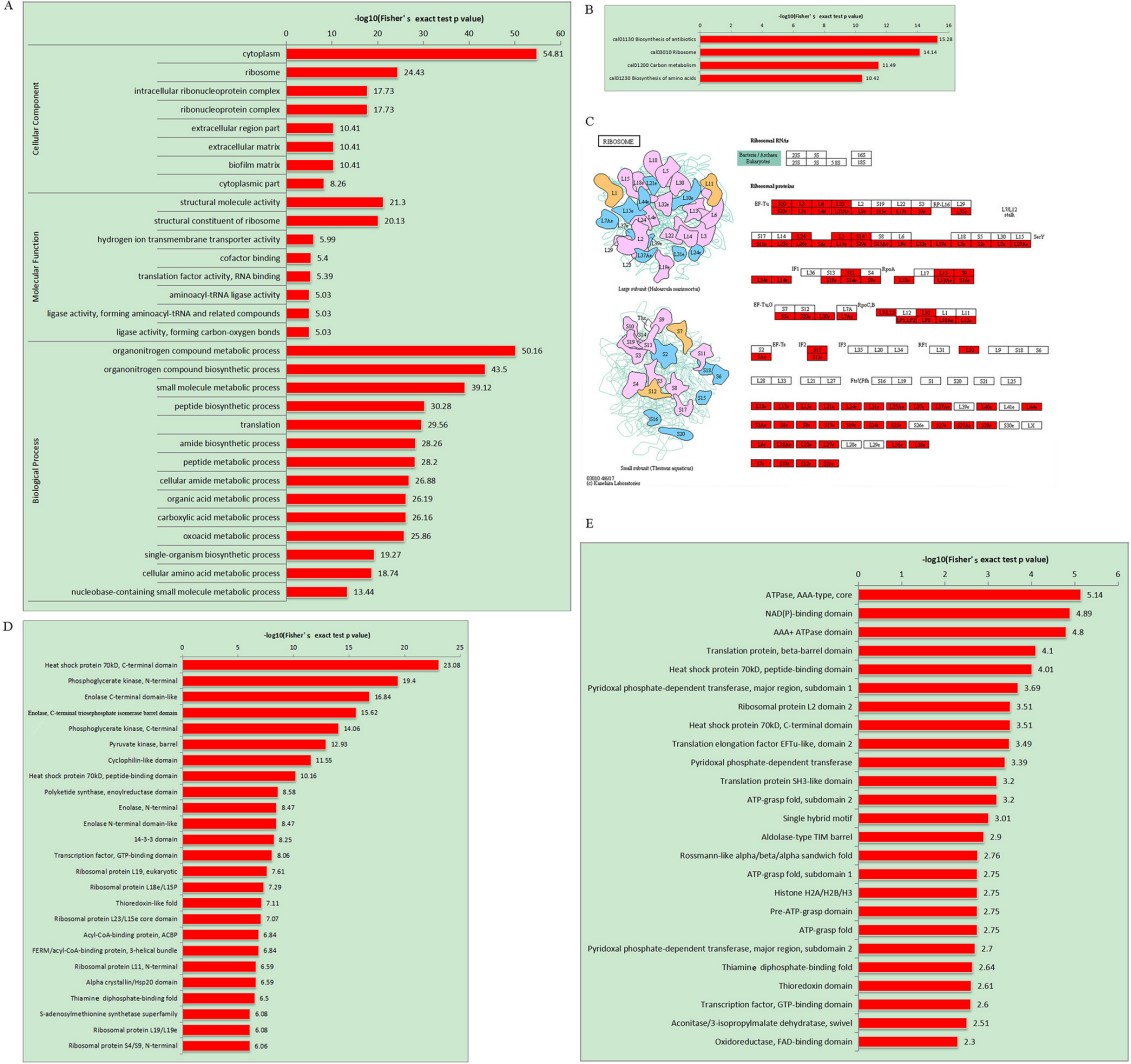
Functional enrichment analysis of lysine crotonylation in C. albicans. (A) GO-based enrichment analysis of identified proteins. (B) KEGG pathway enrichment analysis of the identified modified proteins. (C) The proteins in red were identified in ribosome of this study. Protein domain enrichment analysis of identified sites (D) or proteins (E).

Additionally, annotated proteins from the functional analysis above were tested by Fisher’s exact test to determine whether the enriched functions and pathways are significant according to their *P* value in functional enrichment analysis under the GO categories of cellular component, biological process, and molecular function. We found that crotonylated proteins are markedly involved in ribosomal biogenesis, protein translation, and metabolic processes. As shown in Fig. 3A and Table S4, cytoplasm, ribosome, intracellular ribonucleoprotein complex, and ribonucleoprotein complex are significantly enriched among these crotonylated proteins, which are heavily involved in biological processes such as organonitrogen compound metabolism and biosynthesis, small molecule metabolism, amide biosynthetic process, translation, peptide metabolism, and biosynthesis. It seems that crotonylated proteins are related to hydrogen ion transmembrane transporter activity or to structural constituent molecular complexes such as ribosomes. It is perhaps significant that we find, in addition to gross ribosome structure functions, significant enrichment of cofactor binding, aminoacyl-tRNA ligase activity, translation factor activity (RNA binding), and aminoacyl-tRNA- and other-related-compound-forming ligase activity (e.g., forming carbon-oxygen bonds) to complete protein translation.

10.1128/mSystems.01316-20.4 TABLE S4 GO_enrichment. Download Table S4, XLSX file, 6.1 MB. Copyright © 2021 Zhou et al. 2021 Zhou et al. This content is distributed under the terms of the Creative Commons Attribution 4.0 International license.

With KEGG pathway enrichment analysis of the identified crotonylated proteins, we found that a majority of these proteins are involved in antibiotic biosynthesis, amino acid biosynthesis, and ribosomal biogenesis and carbon metabolism as shown in Fig. 3B and Table S5. The domain enrichment analyses carried out against the identified proteins (Fig. 3D) and crotonylation sites (Fig. 3E) resulted in significant contributions of crotonylated proteins to ribosome, energy production, and metabolism.

10.1128/mSystems.01316-20.5 TABLE S5 KEGG_pathway_enrichment. Download Table S5, XLSX file, 0.2 MB. Copyright © 2021 Zhou et al. 2021 Zhou et al. This content is distributed under the terms of the Creative Commons Attribution 4.0 International license.

The ribosome in all living cells is a complex molecular machine for protein synthesis; however, some subunits of the ribosome may have additional functions. Ribosomal protein L6 (RPL6) has been associated with drug resistance in leukemia cells by interfering in apoptosis (26). In addition, the ribosomal protein L24 seems to play a vital role in drug tolerance in human liver cancer HepG2 cell line (27). In this study, we found that 542 crotonylation sites are associated with 89 ribosomal proteins with variable modified sites. Of these proteins, 38 are 60S subunits, which include L8B, L4B, L20, L6, L3, L2A, L10, L24, etc., and 30 are 40S subunits, which are S7, S4, S3, S9B, S1, S15, S6, S17B, S18B, S20, and S12 (Fig. 3C and Table S5).

Domain enrichment identified 534 crotonylation sites involved in carbon metabolism and 478 crotonylation sites involved in amino acid biosynthesis. For example, 40 crotonylation sites are found in phosphoglycerate kinase (PGK), 31 are found in enolase 1, and 25 are found in Adh1p. Other proteins that are involved in glycolysis with a similar pattern of crotonylation sites include pyruvate kinase, phosphoglycerate dehydrogenase (PHGDH), fructose-bisphosphate aldolase, mitochondrial aconitate hydratase, glucose-6-phosphate isomerase, glyceraldehyde-3-phosphate dehydrogenase, pyruvate decarboxylase, transaldolase, 6-phosphogluconate dehydrogenase, acetyl-CoA carboxylase, pyruvate dehydrogenase E1 component subunit alpha, and subunits of mitochondrial succinate dehydrogenase. Some of these proteins carry multiple functions in metabolism. For example, pyruvate kinase is involved in carbon metabolism, biosynthesis of amino acids, and biosynthesis of antibiotics. PHGDH acts on serine biosynthesis, and the inhibition of PHGDH was proposed to treat epidermal growth factor receptor-tyrosine kinase inhibitor (EGFR-TKI)-resistant lung adenocarcinoma (28). The deficiency of PGK (key enzyme of glycolysis) leads to various central nervous system disorders, myopathy, and nonspherocytic hemolytic anemia in humans (29). PAS domain-containing PGK (LmPAS-PGK) demonstrates the effect of PGK on cell survival through autophagy in *Leishmania* (30).

Moreover, the metabolism of cofactors required for glycolysis, amino acid metabolism, and stress response proteins is also modified by crotonylation as shown in Table S5 and Fig. 3D and 3E. These proteins contain heat shock protein (HSP) 70kD domain, NAD(P)-binding domain, beta-barrel domain, ATPase domain, peptide-binding domain, thiamine diphosphate-binding fold, thioredoxin domain, transcription factor, GTP-binding domain, pyridoxal phosphate-dependent transferase domain, flavin adenine dinucleotide (FAD)-binding domain, translation protein SH3-like domain, and translation elongation factor EFTu-like domain as shown in Fig. 3E and Table S6, ident-proteins. In general, domain analysis shows a predominance of ATPase (AAA-type, core) domain when run against crotonylated proteins, and the result switches to show a predominance of C-terminal domain of heat shock protein (HSP) 70kD when run against crotonylation sites (Fig. 3D, Table S6, ident-sites).

10.1128/mSystems.01316-20.6 TABLE S6 Protein domain enrichment. Download Table S6, XLSX file, 0.6 MB. Copyright © 2021 Zhou et al. 2021 Zhou et al. This content is distributed under the terms of the Creative Commons Attribution 4.0 International license.

### The exploration of lysine crotonylated motifs.

**FIG 4:**
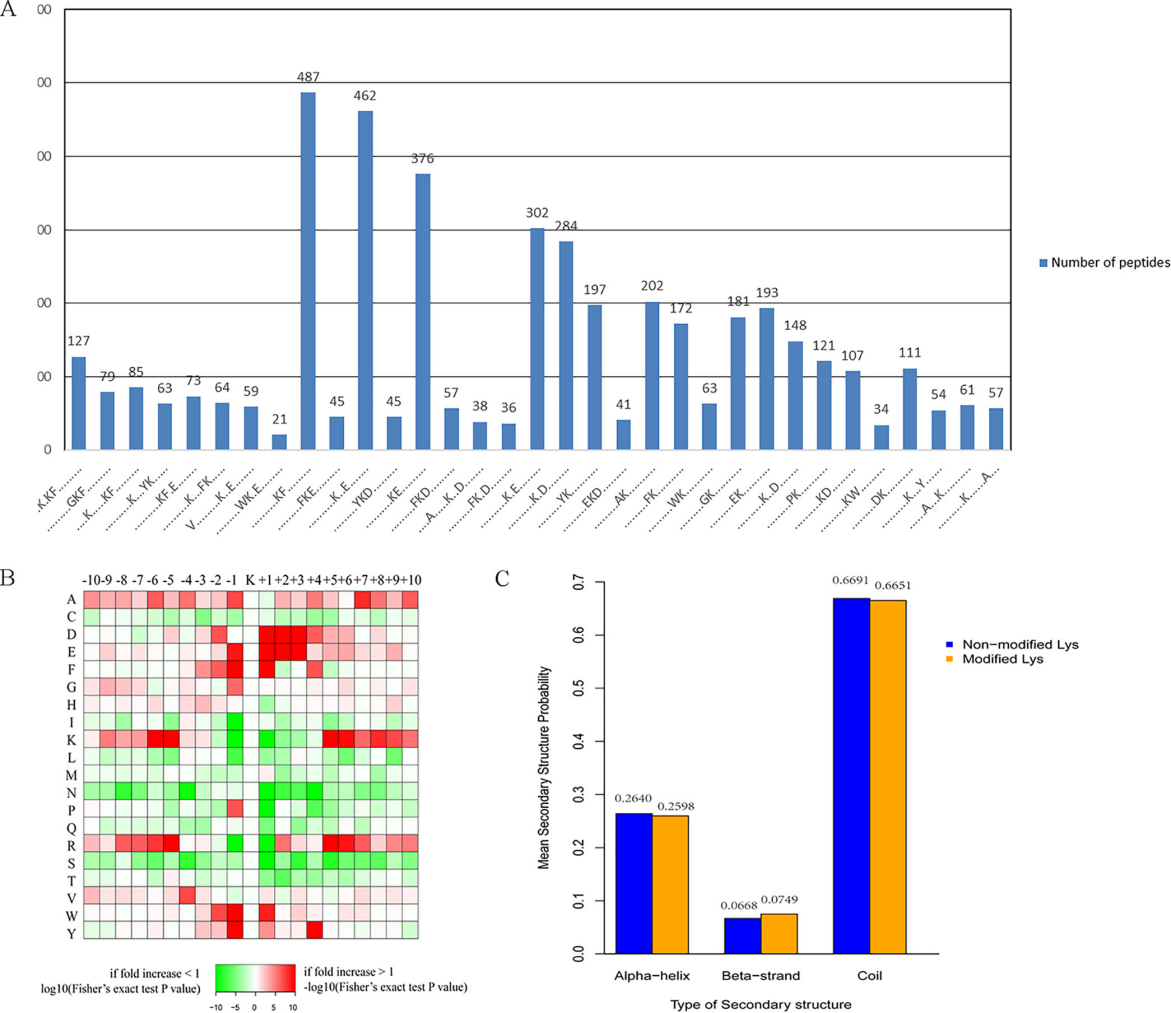
Motif analysis of all identified crotonylated sites. (A) The statistics of motifs involving identified peptides with crotonylated sites. (B) Heat map showing the periodicity of the diverse types of amino acids nearby the crotonylated lysine. (C) Statistics of secondary structures involving identified lysine crotonylated sites. The contrast of a variety of secondary structures (α helix, β strand, and coil) involving identified crotonylated lysine and all lysine secondary structures.

A total of 4,446 Kcr peptides from all identified peptides with amino acids around the crotonylated lysine from the −10 to +10 positions are subjected to the motif-x program (31) for determination of crotonylated motifs. The crotonylated lysine contexts generated 33 conserved motifs as seen in Table 2 and Table S7. The top six motifs and their abundances are as follows: **********KcrF********* (487 peptides), **********Kcr**E******* (462 peptides), **********KcrE********* (376 peptides), **********Kcr*E******** (302 peptides), **********Kcr*D******** (284 peptides), and *********AKcr********** (202 peptides), where K represents lysine, A alanine, F phenylalanine, E glutamic acid, and D aspartic acid, respectively, and “*” indicates any arbitrary single amino acid residue. The data are given in their entirety in Fig. 4A and Table S7.

**TABLE 2:**
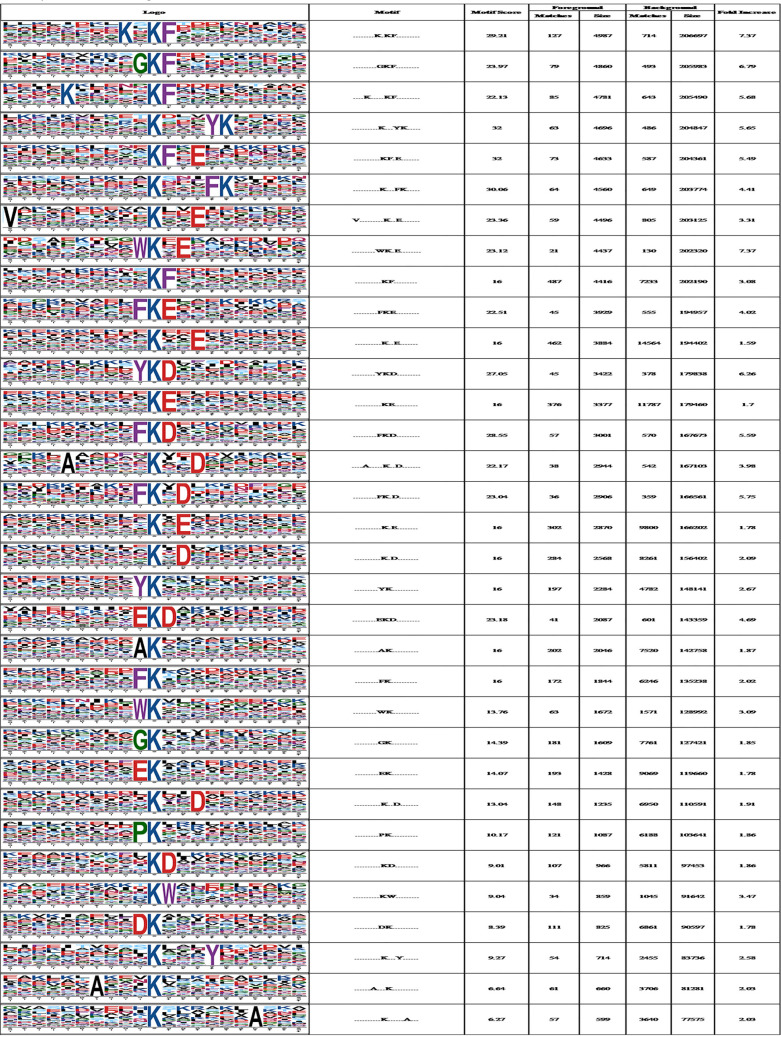
The analysis of enriched crotonylated sites in particular positions (10 amino acids close to two sides of the crotonylated site) through motif-x software

10.1128/mSystems.01316-20.7 TABLE S7 Motif analysis. Download Table S7, XLSX file, 0.2 MB. Copyright © 2021 Zhou et al. 2021 Zhou et al. This content is distributed under the terms of the Creative Commons Attribution 4.0 International license.

Both hydrophobic amino acids such as alanine (A), phenylalanine (F), tryptophan (W), and tyrosine (Y) and acidic amino acids such as glutamic acid (E) and aspartic acid (D) are located near the crotonylated lysine, particularly in areas with a high occurrence of A, D, E, F, glycine (G), histidine (H), valine (V), W, or Y. Usually, D or E is at position +1, +2, or +3, with R or K at +5 or +6. At position −1, the amino acid can be A, E, F, G, P (proline), W, or Y, but at positions −5, −6, −7, or −8 R, K or A is highly present (Fig. 4B and Table S7). Crotonylation should neutralize the positive charge of the Lys residue that increases the hydrophobicity of modified histone. Meanwhile, this change also compromises the ability of the Lys side chain to electrostatically interact with negatively charged molecules such as DNA and acidic amino acid residues (D and E). The overall protein secondary structures in all crotonylation sites show that 25.98% of the sites contain α-helix, 66.5% contain coil structure, and 7.49% contain β-sheet structure (Fig. 4C), which are similar to those found in acetyl proteome in C. albicans (13).

### The crotonylated lysine in histones.

**FIG 5:**
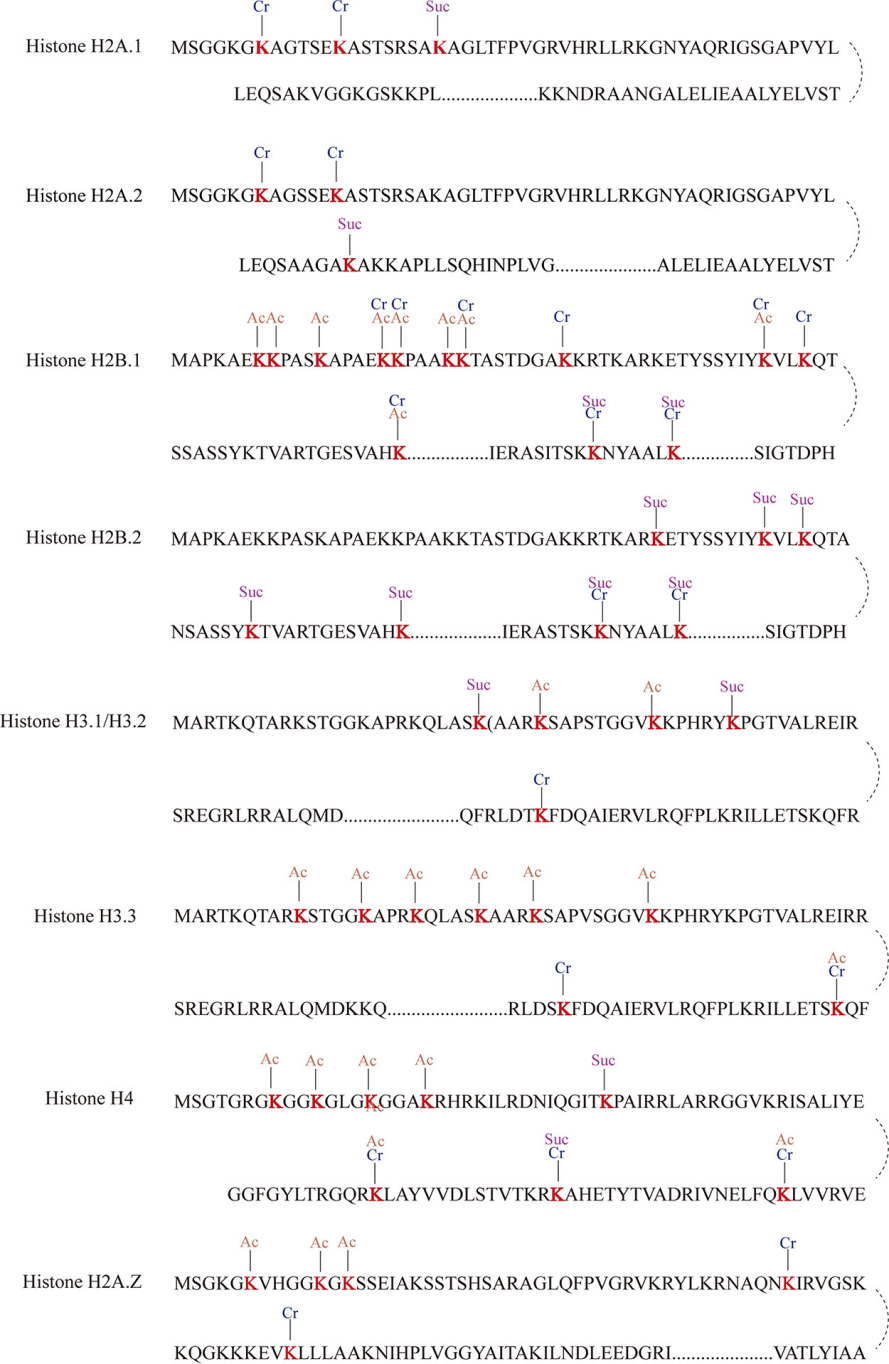
Kac, Ksuc, and Kcr sites on histones of C. albicans identified in this study and our previous data.

The PTM of histone plays a vital role in transcription initiation. Histone H3-K56 acetylation (H3K56ac) has been directly linked to transcriptional activation and DNA preinitiation complex (32–34). H3K56ac has been also associated with pathogenicity of C. albicans (35). The inhibitor of human lysine acetyltransferase KAT6B presents a treatment for small cell lung cancer (36). Bao et al. found that sirt3 regulates histone crotonylation as an “eraser” in cells (24). With anti-Kcr antibody to enrich Kcr peptides in this study, we found that a large number of histone proteins in C. albicans are modified by crotonylation, particularly the histones H2A/H2B/H3. We even note that 23 crotonylation sites appear at tails of C. albicans histones (Table 3 and Table S1). While mapping these sites together with acetylated (13) and succinylated (14) sites on C. albicans histones, 13 of 23 histone tails were simultaneously modified by one of these other modifications. As seen in Fig. 5, H2B.1 (K16, K17, K22, K46, and K111), H3.3 K56, and H4 (K6 and K93) are both modified by acetylation and crotonylation. H2B.1 (K82 and K88), H2B.2 (K82 and K88), and H4K79 are modified by succinylation and crotonylation (5). These double or higher-order modifications imply a more complex level of gene regulation even through this “single” epigenetic mechanism.

**TABLE 3:** Crotonylation sites on histones of C. albicans identified in this study

Protein accession	Position	Amino acid	Protein name	Gene name
H2A				
Q59SU5	7	K	Histone H2A.1	HTA1
Q59SU5	13	K	Histone H2A.1	HTA1
Q59VP2	7	K	Histone H2A.2	HTA2
Q59VP2	13	K	Histone H2A.2	HTA2
Q5AEE1	48	K	Histone H2A.Z	HTZ1
Q5AEE1	125	K	Histone H2A.Z	HTZ1
H2B				
P48989	17	K	Histone H2B.1	HTB1
P48989	18	K	Histone H2B.1	HTB1
P48989	23	K	Histone H2B.1	HTB1
P48989	31	K	Histone H2B.1	HTB1
P48989	47	K	Histone H2B.1	HTB1
P48989	50	K	Histone H2B.1	HTB1
P48989	83	K	Histone H2B.1	HTB1
P48989	89	K	Histone H2B.1	HTB1
P48989	112	K	Histone H2B.1	HTB1
Q59VP1	83	K	Histone H2B.2	HTB2
Q59VP1	89	K	Histone H2B.2	HTB2
H3				
Q59VN2	80	K	Histone H3.1/H3.2	HHT21
Q5ADQ0	57	K	Histone H3.3	HHT3
Q5ADQ0	80	K	Histone H3.3	HHT3
H4				
Q59VN4	62	K	Histone H4	HHF1
Q59VN4	80	K	Histone H4	HHF1
Q59VN4	94	K	Histone H4	HHF1

### Enrichment clustering analysis of multiple PTM modified proteins.

**FIG 6:**
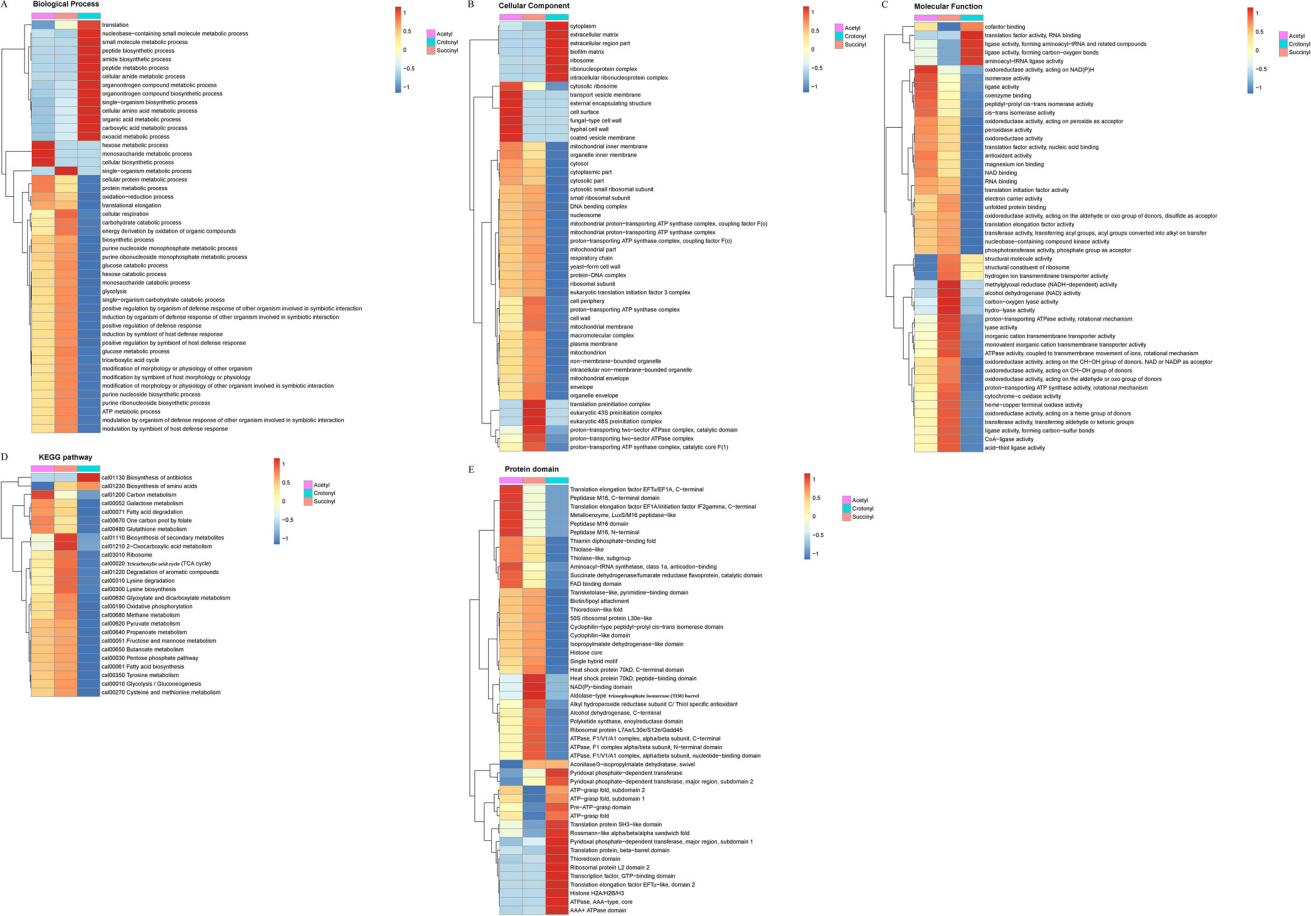
Enrichment analysis of cross talk between lysine acetylation, succinylation, and crotonylation on biological process (A), cellular component (B), molecular function (C), KEGG pathway (D), and protein domain (E).

In our previous studies, we have crossed lysine acetylation with phosphorylation (13) and lysine succinylation with phosphorylation (14) at cross talk enrichment analysis. In order to arrive at a better understanding of the impact of these three PTMs on cellular processes, we performed a comprehensive analysis on three sets of modified proteomic data from current crotonylation and earlier acetylation and succinylation studies based on GO, KEGG, and protein domains analysis (Fig. 6).

At the GO platform, 33 (68.75%) of biological processes are affected by acetylation, 32 by succinylation (66.7%), and 30 (62.5%) by both acetylation and succinylation. Fourteen (29.2%) biological processes are affected by crotonylation, which is found with lower levels of overlap with acetylation or succinylation as shown in Fig. 6A. A similar phenomenon was also shown in the cellular compartment category (Fig. 6B). With 40 (80%) singleton acetylation modifications, 37 (74%) singleton succinylation modifications, and 34 (74%) doubleton acetylation and succinylation modifications in cellular compartments, only 7 (14%) are affected by crotonylation modification, again showing less overlap with acetylation and succinylation. In the enrichment of molecular functions (Fig. 6C), cellular activities are involved with 39 acetylation modification (78%), 44 succinylation modifications (88%), and 8 crotonylation modification (16%). Among those 50 cross talk molecular functions, 37 cellular activities are modified by both acetylation and succinylation (74%), which include cofactor binding, hydrogen ion transmembrane transporter activity, structural molecule activity, and structural constituent of ribosome.

We found that 24 pathways are greatly modified by succinylation and acetylation (92%) as well through KEGG enrichment analysis. These pathways are tricarboxylic acid cycle (TCA cycle), carbon metabolism, fatty acid oxidation, glycolysis/gluconeogenesis, ribosome, cysteine, and methionine metabolism. Meanwhile, antibiotic biosynthesis is greatly modified by crotonylation alone. Antibiotic biosynthesis (biosynthesis of secondary metabolites) is controlled by the phosphate signals at the transcriptional and posttranscriptional levels (37, 38). We also note that biosynthesis of amino acids is greatly modified by both crotonylation and succinylation (Fig. 6D).

Domain analysis found 33 (66%) domains with acetylation modification, 27 (54%) domains with succinylation, and 18 (36%) domains with crotonylation. Twenty-three (46%) domains are simultaneously modified by both acetylation and succinylation, and 4 domains (8%) are modified by both acetylation and crotonylation. Moreover, aconitase/3-isopropyl malate dehydratase (swivel) is modified by both succinylation and crotonylation (Fig. 6E).

### PPI network analysis of multiple PTM modified proteins.

**FIG 7:**
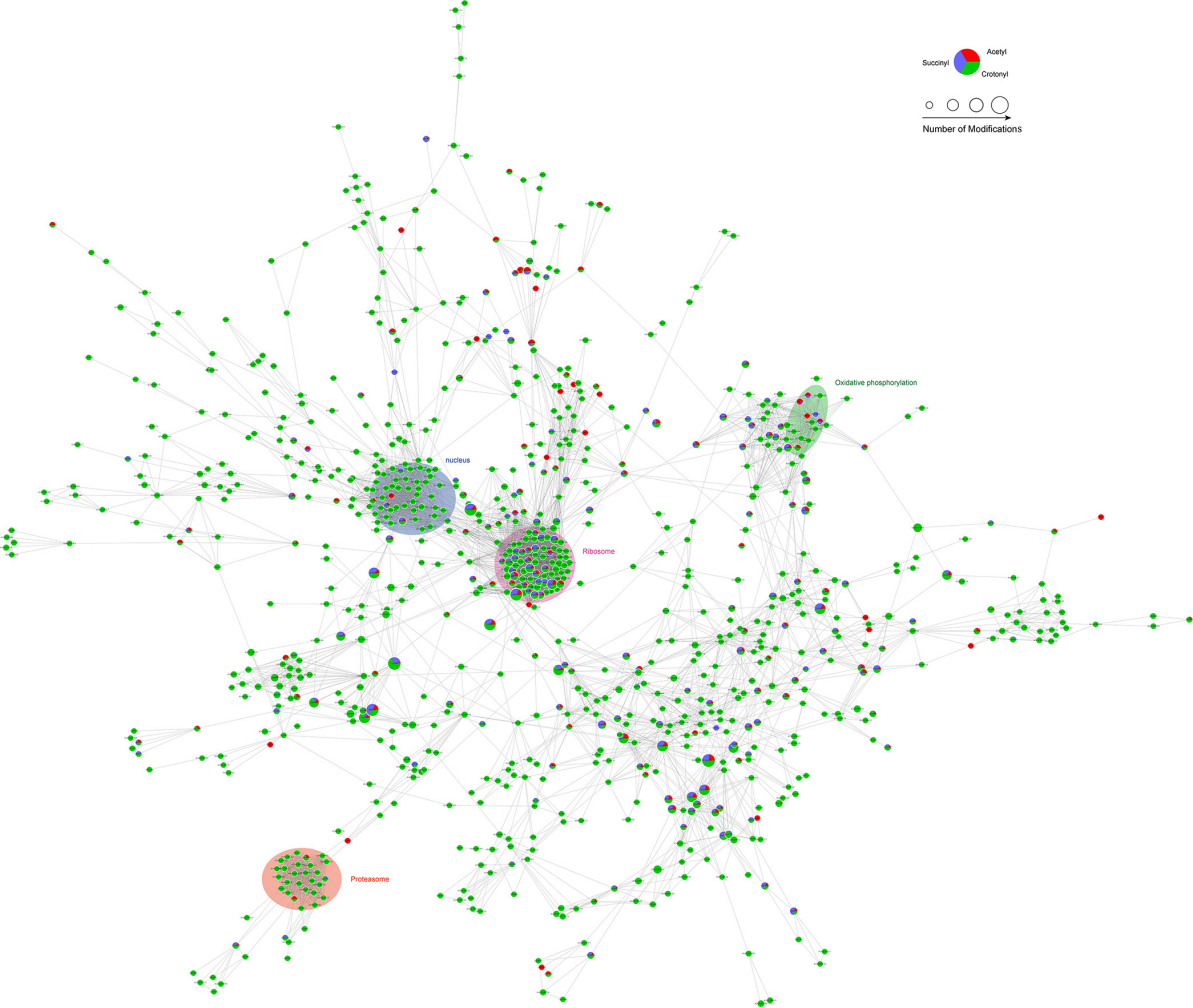
Lysine acetylation, succinylation, and crotonylation of proteins involved in the ribosome, oxidative phosphorylation, nucleus, and proteasome. Crotonylation proteins identified in C. albicans are shown in green ovals, sites that were found to be acetylated are shown as red circles, and succinylated sites are shown as purple circles.

Our previous studies identified 477 acetylated proteins (13) and 389 succinylated proteins (14). Using Cytoscape software, we performed a protein-protein interaction (PPI) analysis to analyze 897 crotonylated proteins, resulting in 60 clusters. The inclusion criteria to choose “finally analyzed” proteins are based on the following: (i) detected proteins with insufficient confidence are filtered out, retaining only those with a confidence score ≥0.9 (highest confidence); (ii) proteins not found to have any interaction with other proteins are filtered out; and (iii) proteins not matched to the corresponding genes in the string database are filtered out. This functional and physical interactions analysis on the STRING database includes all 897 crotonylated proteins as nodes. The PPI interaction among crotonylated proteins is shown in Fig. 7, and the MCODE score defines an advantageous indicator of the PPI network of modified proteins. Within these 897 crotonylated proteins, all 177 proteins simultaneously modified by acetylation and succinylation are included. In generating the PPI network, 136 proteins are simultaneously modified by three PTMs. The most highly modified 20 proteins are as follows: RPL3, RPL5, RPL9B, RPL10A, RPL10, RPL12, RPL13, RPL15A, RPS0, RPS5, RPS20, RPS21, RPS22A, and RP10. These ribosomal proteins are classified in cluster 1 in Fig. 7. Phosphofructokinase PFK1 and PFK2 are classified in cluster 6. Enzymes IMH3 and ADE12 in metabolic pathways (cal01100) are grouped in cluster 10. For mitochondrial energy production, 42 crotonylated proteins are classified in oxidative phosphorylation under cluster 4, cluster 9, cluster 15, and cluster 42, including ATP7 under cluster 15. This protein is F-type H^+^-transporting ATPase subunit in oxidative phosphorylation (cal00190) and metabolic pathways (cal01100). Among these mitochondrial proteins, 10 proteins are modified by acetylation, crotonylation, and succinylation. As for other cellular processes, 25 crotonylated proteins are involved in proteasome (cluster3), of which 2 proteins are modified by both crotonylation and acetylation and 1 protein is modified by both crotonylation and succinylation. Moreover, 19 out of a total of 331 crotonylated proteins are modified by acetylation and succinylation as well, and 21 out of 83 crotonylated proteins are related to protein-protein interaction with three PTMs at the same time (Fig. 7 and Table S8). These results also suggest that crotonylated proteins may play an important role in a variety of cellular processes, as seen in acetylation in eukaryotic cells (39) and E. coli (40).

10.1128/mSystems.01316-20.8 TABLE S8 PTM summary of double and higher-order modifications. Download Table S8, XLSX file, 1.2 MB. Copyright © 2021 Zhou et al. 2021 Zhou et al. This content is distributed under the terms of the Creative Commons Attribution 4.0 International license.

## DISCUSSION

This is the first study of the C. albicans crotonylome. We identified 1,584 crotonylated proteins having a total of 5,242 crotonylation sites during the exponential growth phase of the SC5314 strain under a rich nutrient medium (yeast extract-peptone-dextrose [YPD]). The functional annotation and pathways of the identified crotonylated proteins, together with earlier published acetylome and succinylome data from our group (13, 14), give us a broad understanding of the biological significance of the three PTMs in this organism. Using the same approach to compare the crotonylome to the acetylome or succinylome, we found the number of crotonylated proteins to be 2- to 3-fold higher than we saw for the other two PTMs in C. albicans. We note in particular that there are only 177 crotonylated proteins simultaneously modified by the other two PTMs. The PPI network analysis based on 897 crotonylated proteins also shows a lower level of overlap of crotonylated proteins with acetylated or succinylated proteins than that which was seen between acetylated and succinylated proteins in each GO category and KEGG pathway analysis. These results thus suggest some biological features of proteins under crotonylation modification that will never be seen under the other PTMs.

Functional overlap in all three PTMs is found primarily in ribosomal biogenesis, TCA, mitochondrial oxidative phosphorylation, heat shock adaptation, and the proteasome. However, some antioxidant proteins such as TRX1 (thioredoxin, A0A1D8PU69) and thioredoxin-like proteins orf19.3920 (IPR013766) and PDI (IPR013766) carry only crotonylated modification. Thioredoxin regulates C. albicans responses to H_2_O_2_ stress (41) that is crotonylated in 3 sites (positions 42, 51, and 96). Similarly, we find that glutamate dehydrogenase (GDH3, A0A1D8PMH8) is modified by crotonylation in 2 sites (positions 33 and 402). This enzyme is important in glutamate biosynthesis from ammonia. The deacetylase SIRT4 can effectively decrease activity of glutamate dehydrogenase in mitochondria of mouse pancreatic β cells (42). Whether crotonylation or decrotonylation in C. albicans would be affected by SIRT4 is unknown, but the conversion of the active/inactive form of glutamate dehydrogenase is regulated by phosphorylation in Saccharomyces cerevisiae (43).

Most crotonylated histones are H2, H3, and H4 in C. albicans. We found 17 crotonylation sites in histone H2 and 3 each in histones H3 and H4. Two crotonylation sites were found in histone H3.3, and one site (K56) was even modified by both acetylation and crotonylation. In a total of 23 crotonylated histone tails, 13 were simultaneously modified by the other two modifications. Structurally, all histones contain a globular core domain and more relaxed N-terminal and C-terminal tails where lysine residues on both the globular domain and tails can be modified by posttranslational modifications. H3.3 is already well known for its functions in regulating gene expression, DNA replication, and DNA repair. The acetylation of H3K56 (H3K56ac) can decrease C. albicans toxicity in mouse (35), and H3K56ac is modified by the histone acetyltransferase Rtt109, which is important for C. albicans pathogenicity (44). The exact function of crotonylation at the same H3.3K56ac/H3K56ac site remains elusive but marks an interesting area for further study.

In our previous study, H3K9, H3K14, H3K18, H3K27, and H3K36 were modified by acetylation, and H3K23 was modified by acetylation and succinylation. Protein acetylation was posttranslationally modified by histone acetyltransferases/lysine (K) acetyltransferases (HATs/KATs) and histone deacetylases/lysine (K) deacetylases (HDACs/KDACs). In this study, many acetyltransferases for histone or nonhistone substrates are modified by crotonylation. For example, histone acetyltransferase (at position 3,320), histone acetyltransferase type B subunit 2 (at position 181), and histone acetyltransferase type B catalytic subunit (at position 219) all affect the acetylation modification process of histone. The list of nonhistone acetyltransferases under crotonylation includes peptide alpha-*N*-acetyltransferase complex A subunit, acetyltransferase component of pyruvate dehydrogenase complex, RNA cytidine acetyltransferase, homoserine *O*-acetyltransferase, acetyl-CoA C-acetyltransferase, and carnitine *O*-acetyltransferase. Some deacetylases are also subject to crotonylation, for example, histone deacetylase interacting domain/Sin3, glycoside hydrolase/deacetylase, and histone deacetylase complex. Among these nonhistone acetyltransferases, acetyl-CoA *C*-acetyltransferase bears the greatest number of crotonylation sites with 6 sites (at positions of 165, 196, 212, 219, 225, and 230).

Hsp90 in C. albicans was modified by several types of PTM, including phosphorylation (45, 46). We found 41 crotonylation sites in the current study and 3 acetylation sites (13) and 19 succinylation sites (14) in earlier works. This protein is a chaperone protein that assists other proteins to fold properly and also acts as feedback in stress responses (47–50). Hsp90 PPI network reasserted the close linkage of the S. cerevisiae and C. albicans proteomes (51, 52). The phosphorylation of threonine, serine, and tyrosine regulates the function of Hsp90 in both mammals and yeast (45, 46). In S. cerevisiae, the phosphorylation of threonine 22 and tyrosine 24 of Hsp90 is a crucial step to link to cochaperones (53, 54). Hsp90 has also been associated with drug resistance (55), and KDACs inhibited the Hsp90-dependent azole tolerance in S. cerevisiae and C. albicans (56). Furthermore, Hsp90 responds to stress and regulated echinocandins tolerance via the calcineurin signaling pathway in C. albicans (57). The functional determination of heavily crotonylated HSP90 in drug resistance and stress response seems to offer good promise in combating this fungal pathogen.

In this crotonylome study, we have shown that crotonylation is a more prevalent PTM form than acetylation and succinylation in C. albicans. The biological significance of crotonylation seems to be tied most closely to ribosomal biogenesis/protein translation and proteome and carbon metabolism/mitochondrial energy production. It is to be hoped that a deeper functional analysis of this sort in this organism will pull back the veil even further.

## MATERIALS AND METHODS

### Experimental design and statistical rationale.

We used anti-crotonyllysine antibody (PTM-502, Jingjie PTM Bio, China) enrichment and high-resolution liquid chromatography-mass spectrometry (LC-MS/MS analysis) to analyze the crotonylation in one sample of C. albicans. The purpose of the experiment was to detect whether there is crotonylation in C. albicans, and these results should be considered in light of earlier results involving acetylation and succinylation. The results with a corrected *P* value <0.05 are considered significant.

### Strains, growth conditions, protein extraction, and trypsin digestion.

C. albicans strain SC5314 was obtained from American Type Culture Collection as ATCC-MYA-2876, which was originally isolated from a patient with candidemia in 1984 (58). Overnight growth of fungal cells in YPD (2% glucose, 2% peptone, and 1% yeast extract) at 28°C, 220 rpm was diluted 10-fold with fresh YPD. The exponential growth phase was defined as an additional 4 to 6 h of growth at the same temperature and shaking condition and an optical density at 600 nm (OD_600_) that reaches 0.8. The cell culture was kept at 0°C before crude protein extraction. Cells were first centrifuged at about 4000 to 6000 rpm (4°C), and then the pellet was resuspended and washed with PBS three times.

We used liquid nitrogen to grind the yeast cells, which were then dissolved in lysate buffer (1% protease inhibitors, 3 μM Trichostatin A [TSA], 8 M urea, 1% Triton X-100, 2 mM EDTA, 10 mM dithiothreitol [DTT], 50 mM nicotinamide [NAM]) and then sonicated on ice (3 × 30 s at 2-min intervals) with a high-intensity ultrasonic processor (Scientz, China). The lysates were precipitated at 12,000 × *g* for 1 h and stored at 4°C. The collected protein was mixed with 15% TCA and kept at −20°C overnight for protein precipitation. We used cold acetone to wash precipitate three times. Protein was redissolved in 8 M urea, and protein concentration was determined with bicinchoninic acid (BCA) kit (lot number 23225, Thermo Scientific, USA).

The protein preparation was treated with 5 mM DTT at 56°C for 30 min followed by 11 mM iodoacetamide (IAA) for 15 min in the dark. A total of 100 mM tetraethylammonium bromide (TEAB) was then added to dilute the urea, and trypsin at concentrations 1:50 and 1:100 was then used to digest the protein. The resulting tryptic peptides were suspended in immunoprecipitation (IP) buffer (1 mM EDTA, 100 mM NaCl, 0.5% NP-40, 50 mM Tris-HCl, pH 8.0), and anti-crotonyllysine antibody agarose conjugated beads (PTM-503, Jingjie PTM Bio, China) were blended with peptides at 4°C overnight. After washing beads once with IP buffer and double distilled water, we used 0.1% trifluoroacetic acid (TFA) to elute the enriched peptides. Peptides were then dried and desalted by C18 ZipTips (Millipore, USA) treatment.

### Lysine crotonylation analysis by LC-MS/MS.

**LC-MS/MS analysis.** We used solvent A (0.1% formic acid dissolve in 2% acetonitrile [ACN], 98% H_2_O) to dissolve the tryptic peptides from SC5314. The peptides were then separated by EASY-nLC 1000 (Thermo) at 700 nl/min. Gradient solvent B (0.1% formic acid dissolved in 90% acetonitrile) was used in increasing steps from 8% to 23% (36 min) to 23% to 35% (18 min), then held for 3 min at 80%. The liquid chromatography (LC) separation was carried out on a homemade analytical column with integrated spray tip (150 μm inside diameter [i.d.] × 20 cm) packed with 1.9 μm/120 Å ReproSil-Pur C18 resins (Dr. Maisch GmbH, Ammerbuch, Germany).

The peptides were injected into nanospray ion (NSI) source and separated by Q Exactive HF-X (MS/MS, Thermo). The ion source voltage was 2.0 kV. The ion and secondary fragments of peptide were detected and analyzed by Orbitrap. The *m/z* scan range of MS1 was 350 to 1,600 *m/z*. The resolution was 60,000 for MS1 and 15,000 for MS2. Automatic gain control (AGC) was 1 × 10^5^, the signal threshold was 5,000 ions, the maximum injection time was 100 ms, the maximum number of modifications allowed on the peptide was 5, and the exclusion duration time was set to 15 s.

**Database search.** MS2 data were retrieved by Maxquant (v1.5.2.8). Retrieval parameter setting was the database of Candida albicans strain SC5314 in UniProt with 6,035 sequences. The anti-library and the common contamination library were used to calculate the false-discovery rate (FDR). Other parameters were as follows: cleavage enzyme was set as trypsin/P and missing points was set at 4; the mass error tolerance of primary parent ions of first search and main search was 20 ppm and 5 ppm, respectively, and the secondary fragment ions’ mass error tolerance was 0.02 Da; fixed modification was set as cysteine alkylation, and variable modification was set as methionine oxidation, N-terminal acetylation of protein, and crotonylation modification. Protein identification and FDR identified by PSM were set at 1%.

**Bioinformatics analysis. GO annotation.** The annotation of Gene Ontology (GO) regarding cellular component, biological process, and molecular function was analyzed under the UniProt-GOA database (http://www.ebi.ac.uk/GOA/).

### Domain annotation.

The software InterProScan based on the protein sequence algorithm and the InterPro domain database (http://www.ebi.ac.uk/interpro/) were used to annotate the protein domain of the identified modified proteins.

### KEGG pathway annotation.

We used the KEGG online service tool KAAS to analyze and annotate the protein database, and then we used KEGG mapper to match the corresponding pathways.

### Subcellular localization.

We annotated the subcellular location of the identified proteins using WoLF PSORT (a software for predicting subcellular localization of proteins).

### Functional enrichment analysis.

The enrichment analyses for the GO, KEGG pathway, and domains were performed separately. Categories with *P* values <0.05 are considered significant.

### Motif determination.

When a characteristic sequence number exceeds 20 with a *P* value <0.000001, this sequence context is considered to be a motif of crotonylated peptides. We used motif-x to analyze the sequence models constituted with particular amino acids (10 amino acids downstream and upstream around the crotonylated sites).

### Enrichment-based clustering analysis.

After protein enrichment analysis, we used the function *x* = −log_10_(*P* value) to convert the *P* value and one-way hierarchical clustering to cluster the category. The “heatmap.2” function was used to make a visualized heat map.

### Protein-protein interaction network.

We used cytoscape software to visualize the PPI network. The network of protein-protein interactions was obtained from STRING database version 10.5. STRING categories are arranged by confidence score. In this study, we fetched all interactions that had a confidence score ≥0.9 (highest confidence).

### Data availability.

The mass spectrometry proteomics data have been deposited to the ProteomeXchange Consortium via the PRIDE (59) partner repository with the data set identifier PXD018848, and annotated spectra of identified peptides can be browsed at MSViewer with accession number sddd5ecp39.
